# Analysis of Variation in Organizational Definitions of Primary Care Panels: A Systematic Review

**DOI:** 10.1001/jamanetworkopen.2022.7497

**Published:** 2022-04-15

**Authors:** Michael F. Mayo-Smith, Rebecca A. Robbins, Mark Murray, Rachel Weber, Pamela J. Bagley, Elaina J. Vitale, Neil M. Paige

**Affiliations:** 1 Dartmouth Geisel School of Medicine, Hanover, New Hampshire; 2 Harvard Medical School Center for Primary Care, Boston, Massachusetts; 3 Dartmouth Hitchcock Medical Center, Lebanon, New Hampshire; 4 Mark Murray and Associates, Sacramento, California; 5 Healthcare IE LLC, Mission Viejo, California; 6 VA Greater Los Angeles Healthcare System, Los Angeles, California; 7 David Geffen School of Medicine, University of California, Los Angeles

## Abstract

**Importance:**

Primary care panel size plays an increasing role in measuring primary care provider (ie, physicians and advanced practice providers, which include nurse practitioners and physician assistants) workload, setting practice capacity, and determining pay and can influence quality of care, access, and burnout. However, reported panel sizes vary widely.

**Objective:**

To identify how panels are defined, the degree of variation in these definitions, the consequences of different definitions of panel size, and research on strengths of different approaches.

**Evidence Review:**

Following Preferred Reporting Items for Systematic Reviews and Meta-analyses (PRISMA) guidelines, MEDLINE, Web of Science, Embase, and Dissertations and Theses Global databases were searched from inception to April 28, 2021, for subject headings and text words to capture concepts of primary care panel size. Article review and data abstraction were performed independently by 2 reviewers. Main outcomes reported included rules for adding or removing patients from panels, rules for measuring primary care provider resources, consequences of different rules on reported panel size, and research on advantages and disadvantages of different rules.

**Findings:**

The literature search yielded 1687 articles, with 294 potentially relevant articles and 74 containing relevant data. Specific practices were identified from 29 health care systems and 5 empanelment implementation guides. Patients were most commonly empaneled after 1 primary care visit (24 of 34 [70.6%]), but some were empaneled only after several visits (5 [14.8%]), enrollment in a health plan (4 [11.8%]) or any visit to the health care system (1 [3.0%]). Patients were removed when no visit had occurred in a specified look-back period, which varied from 12 to 42 months. Regarding primary care provider resources, half of organizations assigned advanced practice providers independent panels and half had them share panels with a physician, increasing the physician’s panel by 50% to 100%. Analyses demonstrated that changes in individual rules for adding patients, removing patients, or estimating primary care provider resources could increase reported panel size from 20% to 100%, without change in actual primary care provider workload. No research was found investigating advantages of different definitions.

**Conclusions and Relevance:**

Much variation exists in how panels are defined, and this variation can have substantial consequences on reported panel size. Research is needed on how to define primary care panels to best identify active patients, which could contribute to a widely accepted standard approach to panel definition.

## Key Points

**Question:**

Is there variation in how organizations define primary care panels, and if so, how much does this contribute to variation in reported panel size?

**Findings:**

This systematic review of 74 articles reporting on 29 health care systems and 5 empanelment implementation guides found wide variation in the rules used to add and remove patients from primary care panels and to count primary care resources. Different rules were associated with large differences in reported panel size, independent of the actual clinician workload.

**Meaning:**

These findings suggest that caution is needed comparing reported panel sizes, and research is needed on the benefits of different approaches.

## Teaser

This systematic review identifies how primary care panels are defined, the degree of variation in these definitions, the consequences of different definitions of panel size, and research on strengths of different approaches.

## Introduction

As panel size plays an increasing role in measuring the workload of primary care providers (PCPs), ie, primary care physicians and advanced practice providers (APPs), which include nurse practitioners and physician assistants; setting limits on practice capacity; and determining pay, it has become an issue of much interest to both practice managers and PCPs.^1,2,3^ The association of panel size with outcomes including quality of care, access, and PCP burnout has also received research attention, sometimes with the goal of identifying an optimal panel size.^4^

Looking to the literature for what might be a suitable panel size, one finds marked differences in reported size. For example, Board of Family Medicine applicants in full-time practice estimated their panels to be from fewer than 500 to more than 5000 patients.^5^ While patient characteristics are associated with demand for care and the type and quality of practice support are associated with productivity,^2,4^ it is hard to reconcile these factors alone with the enormous variation in reported panel size. What has been overlooked in these discussions is the lack of an established, standardized approach to defining panels. A recent analysis showed that for one hypothetical panel, the reported panel size could vary from 700 patients to more than 5000 patients, depending on which rules to define a panel are used.^6^

While panel sizes have been widely reported, there has been little exploration of how panels are defined. We undertook a systematic review of medical literature to identify (1) how organizations and researchers define a primary care panel, (2) variation in this definition, (3) consequences of this variation on reported panel size, and (4) research on strengths or weaknesses of specific measurement approaches.

## Methods

### Data Sources and Searches

We performed this review in accordance with Preferred Reporting Items for Systematic Reviews and Meta-analysis (PRISMA) 2020.^7^ Research librarians with expertise in systematic reviews developed and conducted searches for English-language studies from the date of inception to April 28, 2021, in MEDLINE (Ovid), Web of Science (Clarivate Analytics), Embase (Ovid), and Dissertations and Theses Global (ProQuest). The search included subject headings and text words to capture the concepts of primary care and panel size. The search strategy was adjusted for the syntax appropriate to each database (eTable 1 in the Supplement).

A gray literature search was done to identify documents produced by medical associations about panel size. A site search for panel size was conducted on the websites of the following organizations: American Medical Association, American Academy of Family Physicians, American Academy of Pediatrics, Canadian Medical Association, and College of Family Physicians of Canada. The first 10 references listed were reviewed for relevance.

### Study Selection

Table 1 provides definitions of the terms related to primary care panel as used in this article. Titles and abstracts were reviewed in duplicate by 2 authors (M.M.S. and R.A.R.). We selected articles for full text review that might include description of rules used to define primary care panel or included primary care panel size as independent or dependent variable. We limited articles to reports from United States and Canada.

**Table 1.** Definition of Terms Related to Primary Care Panels

Term	Definition
Empanelment	The process of assigning each patient in a primary care practice to a specific primary care provider (physician or advanced practice provider).
Panel	A group of patients that has been empaneled to a specific primary care provider.
Panel size	No. of patients in a given panel.
Primary care provider resources	Amount of primary care provider resources (clinical full-time equivalent physician and/or advanced practice provider) assigned to care for a given panel.
Panel capacity	Target panel size for a given primary care provider in a specific practice. This will vary depending on patient, practice, and primary care provider characteristics.

### Data Abstraction

Full text review was conducted independently in duplicate by at least 2 authors (M.M.S., R.A.R., M.M., R.W., or N.M.P.), using a standard, piloted data extraction form (eMethods in the Supplement). Discrepancies were resolved through discussion. Articles were included if they contained any description of (1) rules for adding or removing patients from panels, (2) rules for measuring PCP resources, (3) consequences of different rules on reported panel size, or (4) research on advantages and disadvantages of different approaches to defining panel size.

### Assessing Risk of Bias

As the primary outcome was a description of organizational practices, without experimental intervention, we felt the risk of bias in individual studies was minimal. We did not synthesize findings from numerous studies into a single summary. Use of published reports rather than representative surveys introduced a risk that the distribution of findings might vary from distribution across all health care delivery organizations. We accepted this risk as inherent in the study design and acknowledged it as a limitation in our discussion.

### Statistical Analysis

A 2-tailed *t* test was used to test for differences between mean of 2 groups and Pearson *r* for correlation. Analyses were conducted in Excel Statistics version 2202 (Microsoft Corp).

## Results

### Literature Search

**Figure 1.**
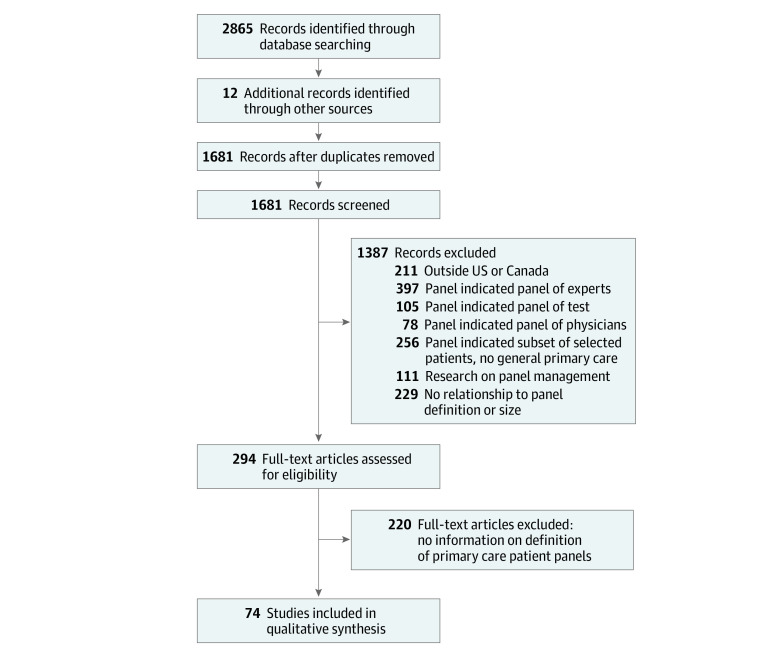
Study Flowchart

As shown in Figure 1, the literature search yielded 1671 articles. Title and abstract review identified 294 potentially relevant articles that underwent full text review, with 74 including relevant data.^8,9,10,11,12,13,14,15,16,17,18,19,20,21,22,23,24,25,26,27,28,29,30,31,32,33,34,35,36,37,38,39,40,41,42,43,44,45,46,47,48,49,50,51,52,53,54,55,56,57,58,59,60,61,62,63,64,65,66,67,68,69,70,71,72,73,74,75,76,77,78,79,80,81^

### Patient Assignment

Table 2 shows the findings for rules for adding and removing patients from panels and the frequency of updating panel lists as reported by 29 different health care systems and 5 empanelment guides.^8,9,10,11,12,13,14,15,16,17,18,19,20,21,22,23,24,25,26,27,28,29,30,31,32,33,34,35,36,37,38,39,40,41,42,43,44,45,46,47,48,49,50^ Details for each system or guide is provided in eTable 2 in the Supplement. Most organizations (29 [85.3%]) added patients to panels after 1 or more visits to primary care occurred (24 [70.6%] after 1 visit and 5 [14.8%] after several). However, 4 (11.8%) empaneled patients at the time of enrollment into a health plan regardless of whether they had received care, and 1 (3.0%) included patients after any visit to the health care system. Most organizations removed patients from panels based on a look-back period without utilization of primary care. As shown in Table 2, this ranged from 12 months to 36 months. One reported removing patients when they had no utilization of services of any type from the organization in 42 months, and 1 when they disenrolled from their health plan. Two also removed patients when notified of their death. Practices reported updating panels at intervals ranging from twice monthly to annually.

**Table 2.** Summary of Findings From 29 Different Health Care Systems and 5 Empanelment Implementation Guides^a^

Criteria	No./total No. of systems or guides (%)
Criteria for assignment to panel	
Visit to primary care	
1	24/34 (70.6)
2-3	5/34 (14.7)
Health plan enrollment	4/34 (11.8)
Any visit to delivery system, and reside locally	1/34 (2.9)
Criteria for removal from panels	
No visit in 12 mo	4/28 (14.3)
No visit in 18 mo	8/28 (28.6)
No visit in 24 mo	4/28 (14.3)
No visit in 36 mo	8/28 (28.6)
No visit in 42 mo	1/28 (3.6)
Health plan disenrollment	1/28 (3.6)
Death (occurring before other end points)	2/28 (7.1)
Frequency of updating panel assignment	
Twice monthly	1/18 (5.6)
Monthly	9/18 (50.0)
Every 3 mo	4/18 (22.2)
Every 6 mo	1/18 (5.6)
Annually	3/18 (16.7)
APPs	
APPs have independent panels	7/17 (41.2)
APPs and physician share panels	7/17 (41.2)
System uses both models	3/17 (17.6)
Substitution ratio for APP	
1.00	2/7 (28.6)
0.80	1/7 (14.3)
0.75	3/7 (42.9)
0.50	1/7 (14.3)

Abbreviation: APP, advanced practice provider.

^a^ Totals for each item vary as not all articles reported every item.

### PCP Resources

As panel size is a ratio of assigned patients to a unit of PCP resources, rules for determining PCP resources also affect panel size. One factor affecting PCP resources is how the organization accounts for the contributions of APPs, which include nurse practitioners and physician assistants.^29,30,31,32,33,34,35,36,37,38,39,40,41,42,43,44,45,46,47,48,49,50,51,52,53,54,55,56,57,58^ APPs can have independent panels; alternatively, patients they care for can be counted in panels of associated physicians. Seven organizations (20.6%) reported establishing independent panels for APPs, 7 (20.6%) reported that APPs shared panels with physicians, and 3 (8.8%) reported using both models. A national survey of a sample of nurse practitioners in 2012 revealed that 64% of those working in primary care reported having their own panels.^54^ In single-state surveys of primary care nurse practitioners from New York and Massachusetts, 42% and 45%, respectively, reported having their own panels.^52,53^

For practices that use APPs, it is common to use a substitution ratio that represents the portion of a physician full-time equivalent (FTE) workload that can be added to practice capacity with the addition of an APP. For example, with a substitution ratio of 0.75, a full-time APP with an independent panel will include 75% as many patients as a physician, or if the APP does not have an independent panel, the physician panel will increase by 75%. The reported substitution ratios ranged from 0.5 to 1.0, as shown in Table 2.^40,41,42,43,44,45,46,47,48,49,50^

Another key consideration in determining PCP resources is adjustment for portion of the PCP’s effort that is dedicated to primary care clinical practice (primary care clinical FTE [CFTE]). We identified 28 articles that address whether panel was adjusted for CFTE or not (eTable 3 in the Supplement).^8,9,10,11,12,16,19,27,31,34,37,38,39,40,41,43,44,45,58,59,60,61,62,63,64,65,66,67^ Of these, 14 (50%) adjusted for the PCPs’ CFTE, but 14 (50%) reported panel size that was not adjusted for PCPs’ CFTE. Twenty-one of these articles (75.0%) also reported average panel sizes.^9,10,11,12,31,37,38,39,40,41,44,45,58,59,60,62,63,64,65,66,67^ The average reported panel size in the reports that did not adjust for CFTE was only 48% the size of the average panel size in those that did adjust for CFTE, a statistically significant difference (*t* = 4.47; *P* < .001) (eTable 4 in the Supplement).

We also identified 11 studies that undertook modeling of hypothetical primary care practices and reported primary care panel sizes as part of their analysis.^68,69,70,71,72,73,74,75,76,77,78^ Five of these (45.5%) estimated the time it would take to provide preventive, acute, or chronic care to a panel of patients; 4 (36.4%) modeled the association of panel size with access; and 2 (18.2%) modeled primary care team mix and productivity. In 10 of these studies (90.9%), panel was conceptualized as a stable pool of patients, all of whom were seen in primary care at least once each year, without consideration of turnover of patients in a primary care panel. One study (9.1%) used a model where the panel was a group of patients who reported they had a regular source of care on the Medical Expenditure Panel Survey, a common method in health services research for identifying patients with a PCP. However, of such patients, only 66% reported visits with their PCP in a given year. Thus, in their hypothetical panel of 2000 patients, only 1313 had a primary care visit in a given year.

### Changes to Panel Size

**Figure 2.**
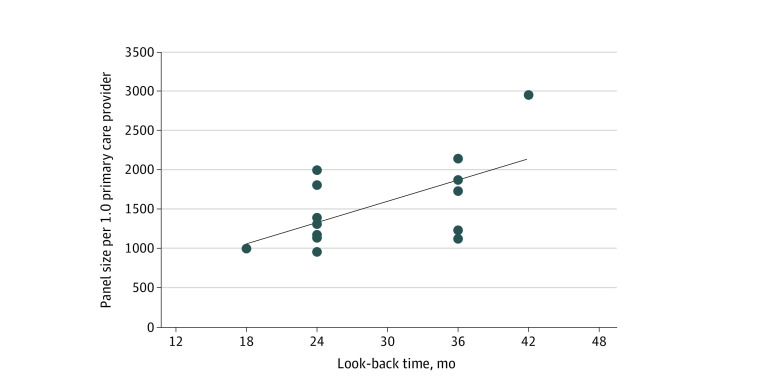
Panel Size per 1.0 Clinical Full Time Employee Primary Care Provider vs Look-Back Period Primary care providers include physicians and advanced practice providers. Look-back period indicates the period a patient remains on the panel without a visit.

Among the 15 organizations that described at least 1 of their rules for defining panel and also reported specific panel sizes per 1.0 CFTE, size ranged from 400 to 2959, with a mean (SD) of 1546 (702) and median (IQR) of 1350 (1132-1944) (eTable 2 in the Supplement).^11,12,13,31,39,40,41,42,43,44,45,56,57,58,59^ We sought data regarding the consequences of different decision rules on reported panel size. We found no published research explicitly examining this topic. However, we did identify articles that provided insights, shown in Table 3. Two articles quantitated the consequences of including patients who do not use primary care services in panels. At Kaiser Permanente Colorado, it was found that panels including all enrollees were 31% larger than panels limited to enrollees who used primary care in the past 18 months.^81^ As described previously, if one looks at Medical Expenditure Panel Survey data, including all patients who identify a PCP leads to a panel that is 52% larger than a panel that is limited to patients who have had a visit with their PCP in the past 12 months.^76^ In addition, studies of patient attrition in 2 delivery systems demonstrated that approximately 1% of patients attrite from their primary care practices each month.^26,35^ Thus, longer look-back periods result in larger panel sizes, increasing approximately 12% for each additional year included in the look-back period, even though actual workload for individual PCPs is not different. We identified practices that reported panel sizes as well as look-back and found a positive correlation between length of look-back and panel size (Pearson *r* = 0.6025; *P* = .02) (eTable 5 in the Supplement).^12,35,37,40,41,42,43,44,45,46,59^ This association is shown in Figure 2. Finally, adding an APP to a practice and counting their contribution in the panel of a physician would lead to reported physician panels that were 50% to 100% larger than those where the APP had their own panel.

**Table 3.** Consequences of Different Panel Rules on Panel Size

Source	Finding		Consequence
	A	B	
**Rules for adding patients to panel**			
Kaiser Permanente Colorado^81^	All enrolled patients: 460 440	Enrolled patients with primary care visit in past 18 mos: 352 009	Panel size 31% larger based on all enrolled patients
Medical Expenditures Panel Survey of patients with usual source of care, sample of 2000^76^	Patients with at least 1 visit to PCP in past 12 mos: 1313	Patients without visit to PCP in prior 12 mos: 687	Panel size 52% larger if patients without PC visit included.
**Rules for removing patients from panel**			
Mayo Clinic, Rochester, Minnesota^35^	10% attrition of patients from primary care between 12 and 24 mos	20% attrition of patients from primary care between 12 and 36 mos	Panel size 20% larger with 36 vs 12-mo look-back.
Beth Israel Deaconess, Boston, Massachusetts^26^	1% attrition of patients from primary care each month	NA	Panel size 24% larger with 36-mo vs 12-mo look-back
**Rules for APPs**			
Hypothetical scenario where 1.0 APP added to practice of 1.0 physician, with substitution ratio of 0.75; baseline physician panel:1000 patients	Independent APP panel: 750	APP patients assigned to physician panel	Panel 75% larger if patients seen by APP assigned to physician panel
	1.0 physician panel:1000	1.0 physician panel: 1750	

Abbreviations: APP, advanced practice provider; NA, not applicable; PCP, primary care provider.

### Research on Different Approaches on Defining Panel and Panel Size

No research was identified investigating the strengths or drawbacks of different rules for defining panel and panel size.

## Discussion

This review found that defining and measuring primary care panels involves several decision points and that there is wide variation in how health care delivery organizations and researchers approach them. Different approaches were associated with significant variation in reported panel size, independent of the number of patients receiving care from a given PCP. Given this finding, much caution is warranted comparing reported panel sizes across organizations and publications, as much of the variation may be because of differing definitions for panel, not differences in actual PCP workload.

Based on these findings, we recommend that when panel composition is an important element of a study, authors at least provide details of how panel was defined and measured. This would include criteria for adding and removing patients, frequency of updating, whether APPs have their own panels, and the substitution ratio used for APPs. Whenever reporting average panel size, it should be adjusted for primary care CFTE and reported as panel size per 1.0 CFTE physician and, when applicable, 1.0 CFTE APP.

The findings also raise the question of what rules lead to a panel list that most accurately captures active primary care patients. Research leading to improved understanding of this question could lay the groundwork for a generally accepted approach to defining panels and measuring panel size. This could reduce the confusion that currently exists about appropriate panel targets, decreasing the risk for both inappropriately large panels, with negative associations with quality, access, and PCP burnout, and inappropriately small panels, with waste of scarce primary care resources. Making panel lists more accurate in their identification of patients who are currently seeking primary care from a PCP or practice might also assist in targeting population management efforts and in assessing quality of care.

The issue of defining and measuring panels is not only important at the local practice level but also has increasing implications for health care policy. Various attribution algorithms have been used to assign individual patients to Accountable Care Organizations, but these attribution algorithms have significant shortcomings.^35^ There is growing interest in moving away from attribution algorithms and toward proactive, explicit linkage of each patient in Medicare to a specific PCP, leading in essence to PCP panels.^82^ Taking this even further, a recent report from the National Academies of Sciences, Engineering and Medicine, *Implementing High-Quality Primary Care*,^83^ advocates for universal empanelment, with each individual linked to a usual source of care, information that would be used for payment and accountability measures. Should this proposal gain traction, primary care assignment and panel size would gain even greater importance, beyond the information they provide to individual delivery systems. Finally, primary care physician supply has been associated with lower mortality at the population level.^84^ Accurate understanding of how many patients can be reasonably cared for by a single PCP is important for right sizing the primary care workforce. The confusion that exists about this basic issue speaks to the need for greater investment in the science of health care operations, especially as primary care in the United States, making up 5% to 7% of a $4 trillion health care enterprise, represents an approximately $200 billion industry.

### Limitations

This study has limitations, including its reliance on data from published literature. The frequency of various approaches seen in published articles may differ from current practices across all organizations, and additional variations in practice may have been missed. Most reports came from academic institutions, which might differ from nonacademic organizations. However, these limitations do not negate the conclusions described. It is also important to appreciate that standardizing measurement rules will not, by itself, resolve the challenges in determining an optimal panel size for individual PCPs. Patients differ in their need for care. Practices differ in their support for PCP productivity. PCPs differ in their professional training and the scope of services covered. Each of these complex issues must be addressed when determining an appropriate workload for individual PCPs. They are additional, important areas for future research. It has been shown for that adjusting panel sizes for patient complexity within a given organization is helpful in balancing workload and improving access.^4^ Nevertheless, having an evidence-based, standard approach to panel definition will support a scientific approach to measuring and addressing these issues in practice. Additionally, it should be noted that the studies examining the association of panel size with outcomes and the adjustment of individual PCP panels for patient characteristics have been done within single organizations.^4^ Thus, the same measurement method was used for all groups within the study, and these analyses remain valid.

## Conclusions

This study found much variation in how different organizations and researchers defined a PCP’s panel, and this variation in rules had substantial consequences for reported panel size. Further research is needed on how to define primary care panels to most accurately capture the list of patients that are currently seeking primary care from a given PCP, which could contribute to a widely accepted, standard approach to panel definition.

## Notes

Supplement. **eTable 1.** Search Strategies for All Databases Searched **eMethods.** Summary of Data Extraction Form **eTable 2.** Details of Panel Definition Rules from 29 Different Health Care Systems and 5 Empanelment Implementation Guides **eTable 3.** Details of Adjustment for Clinical Full Time Employee (CFTE) **eTable 4.** Statistical Analysis of Consequences of Adjustment for CFTE and Panel Size **eTable 5.** Correlation of Panel Size and Duration of Look-Back Period
