# Supplementary material for: Analysis of Variation in Organizational Definitions of Primary Care Panels: A Systematic Review
Source: JAMA Netw Open. 2022 Apr 15;5(4):e227497. doi: 10.1001/jamanetworkopen.2022.7497 (PMC9012968; doi:10.1001/jamanetworkopen.2022.7497)
Supplement: Supplement. — eTable 1. Search Strategies for All Databases Searched eMethods. Summary of Data Extraction Form eTable 2. Details of Panel Definition Rules from 29 Different Health Care Systems and 5 Empanelment Implementation Guides eTable 3. Details of Adjustment for Clinical Full Time Employee (CFTE) eTable 4. Statistical Analysis of Consequences of Adjustment for CFTE and Panel Size eTable 5. Correlation of Panel Size and Duration of Look-Back Period [file jamanetwopen-e227497-s001.pdf]

Supplemental Online Content

Mayo-Smith MF, Robbins RA, Murray M, et al. Analysis of variation in organizational definitions of primary care panels: a systematic review. *JAMA Netw Open.* 2022;5(4):e227497. doi:10.1001/jamanetworkopen.2022.7497

eTable 1. Search Strategies for All Databases Searched

eMethods. Summary of Data Extraction Form

eTable 2. Details of Panel Definition Rules from 29 Different Health Care Systems and 5 Empanelment Implementation Guides

eTable 3. Details of Adjustment for Clinical Full Time Employee (CFTE)

eTable 4. Statistical Analysis of Consequences of Adjustment for CFTE and Panel Size

eTable 5. Correlation of Panel Size and Duration of Look-Back Period

This supplemental material has been provided by the authors to give readers additional information about their work.

© 2022 Mayo-Smith MF et al. *JAMA Network Open.*

eTable 1. Search Strategies for All Databases Searched

Database: Medline (Ovid)
Database(s): **Ovid MEDLINE(R) and Epub Ahead of Print, In-Process, In-Data-Review & Other Non-Indexed Citations and Daily** 1946 to April 27, 2021

#	Searches	Results
1	exp General practice/ or General practitioners/ or Pediatrics/ or Primary Health Care/ or Physicians, Primary Care/ or Pediatricians/ or Physicians, Family/ or (pediatrician* or paediatrician* or family medicine or family practic* or general practic* or Pediatrics or Paediatrics or PCP or primary care*).ti,ab,kf.	365034
2	((empanelment or panel* or roster*) adj4 (patient* or size or workload or primary care or practice or provider or physician)).ti,ab,kf.	5031
3	1 and 2	744
4	limit 3 to english language	726

Database: Web of Science (Clarivate Analytics)
Indexes=SCI-EXPANDED, SSCI, A&HCI, CPCI-S, CPCI-SSH, BKCI-S, BKCI-SSH, ESCI

#3	813	#2 AND #1
#2	8,937	(TS=((empanelment or panel* or roster*) NEAR/4 (patient* or size or Workload or "Primary care" or practice or provider or physician))) AND LANGUAGE: (English)
#1	253,598	(TS=("family medicine" or "family practic*" or "general practic*" or Pediatrics or Paediatrics or PCP or "primary care*" or pediatrician* or paediatrician*)) AND LANGUAGE: (English)

Database: EMBASE (Ovid)
Database(s): Embase 1974 to 2021 April 26

#	Searches	Results
1	exp general practice/ or exp general practitioner/ or pediatrics/ or primary health care/ or pediatrician/	331700
2	(pediatrician* or paediatrician* or family medicine or family practic* or general practic* or Pediatrics or Paediatrics or PCP or primary care*).ab,kw,ti.	352706
3	1 or 2	543449
4	((empanelment or panel* or roster*) adj4 (patient* or size or workload or primary care or practice or provider or physician)).ab,kw,ti.	10044
5	3 and 4	1269
6	limit 5 to english language	1244

Database: Dissertations and Theses Global (ProQuest)

Set	Search	Results
S4	(ti(("family medicine" OR "family practic*" OR "general practic*" OR Pediatrics OR Paediatrics OR PCP OR "primary care*" OR pediatrician* OR paediatrician*)) OR ab(("family medicine" OR "family practic*" OR "general practic*" OR Pediatrics OR Paediatrics OR PCP OR "primary care*" OR pediatrician* OR paediatrician*)) OR su(("family medicine" OR "family practic*" OR "general practic*" OR Pediatrics OR Paediatrics OR PCP OR "primary care*" OR pediatrician* OR paediatrician*))) AND (ti(((empanelment OR panel* OR roster*) NEAR/4 (patient* OR size OR Workload OR "Primary care" OR practice OR provider OR physician))) OR ab(((empanelment OR panel* OR roster*) NEAR/4 (patient* OR size OR Workload OR "Primary care" OR practice OR provider OR physician))) OR su(((empanelment OR panel* OR roster*) NEAR/4 (patient* OR size OR Workload OR "Primary care" OR practice OR provider OR physician)))) Limits applied<br>Databases: ProQuest Dissertations & Theses Global<br>Narrowed by:<br>Language: English	57
S3	(ti(("family medicine" OR "family practic*" OR "general practic*" OR Pediatrics OR Paediatrics OR PCP OR "primary care*" OR pediatrician* OR paediatrician*)) OR ab(("family medicine" OR "family practic*" OR "general practic*" OR Pediatrics OR Paediatrics OR PCP OR "primary care*" OR pediatrician* OR paediatrician*)) OR su(("family medicine" OR "family practic*" OR "general practic*" OR Pediatrics OR Paediatrics OR PCP OR "primary care*" OR pediatrician* OR paediatrician*))) AND (ti(((empanelment OR panel* OR roster*) NEAR/4 (patient* OR size OR Workload OR "Primary care" OR practice OR provider OR physician))) OR ab(((empanelment OR panel* OR roster*) NEAR/4 (patient* OR size OR Workload OR "Primary care" OR practice OR provider OR physician))) OR su(((empanelment OR panel* OR roster*) NEAR/4 (patient* OR size OR Workload OR "Primary care" OR practice OR provider OR physician))))<br>Databases: ProQuest Dissertations & Theses Global	57
S2	ti(((empanelment OR panel* OR roster*) NEAR/4 (patient* OR size OR Workload OR "Primary care" OR practice OR provider OR physician))) OR ab(((empanelment OR panel* OR roster*) NEAR/4 (patient* OR size OR Workload OR "Primary care" OR practice OR provider OR physician))) OR su(((empanelment OR panel* OR roster*) NEAR/4 (patient* OR size OR Workload OR "Primary care" OR practice OR provider OR physician)))<br>Databases: ProQuest Dissertations & Theses Global	697
S1	ti(("family medicine" OR "family practic*" OR "general practic*" OR Pediatrics OR Paediatrics OR PCP OR "primary care*" OR pediatrician* OR paediatrician*)) OR ab(("family medicine" OR "family practic*" OR "general practic*" OR Pediatrics OR Paediatrics OR PCP OR "primary care*" OR pediatrician* OR paediatrician*)) OR su(("family medicine" OR "family practic*" OR "general practic*" OR Pediatrics OR	14,068

© 2022 Mayo-Smith MF et al. *JAMA Network Open.*

	Paediatrics OR PCP OR "primary care*" OR pediatrician* OR paediatrician*))<br>Databases: ProQuest Dissertations & Theses Global	

© 2022 Mayo-Smith MF et al. *JAMA Network Open.*

eMethods. Summary of Data Extraction Form

An Excel spreadsheet was used for data abstraction which included columns for each of the items listed below. Areas for comments were included for each item extracted.

1. Year of publication
2. Reference
3. Article type
4. Institution or setting
5. Definition of "panel"
6. Reported panel size
7. Criteria for assignment to panel
8. Criteria for removal from panel – type of encounter needed to keep patient active*
9. Criteria for removal to panel – duration without qualifying visit
10. Criteria for removal from panel – did report of death lead to removal (yes/no)*
11. Criteria for removal from panel – did move to another site within the same health care system lead to removal?*
12. Frequency of updating panel assignment
13. Panel size adjusted for provider Clinical FTE dedicated to primary care (yes/no)
14. Advanced Practice Providers: independent or shared panel
15. Advanced Practice Providers: Substitution ratio

* Insufficient information on these items to warrant inclusion in results.

© 2022 Mayo-Smith MF et al. *JAMA Network Open.*

eTable 2. Details of Panel Definition Rules from 29 Different Health Care Systems and 5 Empanelment Implementation Guides

Reference	Institution	Reported panel size per 1.0 CFTE MD	Criteria for panel assignment	Criteria for inactivation: duration without visit	Frequency of updating panel assignments	Advanced Practice Providers have own panel	Substitution ratio
**Health Care Delivery Organizations**							
8	George Washington University Health Plan and clinics - HMO patients		Enrollment in health plan for HMO patients, single visit for fee-for-service patients				
9,11	Group Health Cooperative of Puget Sound	2126	Enrollment in health plan	Disenrollment	Quarterly		
10	Massachusetts General Hospital		Single visit	36 months			
41,42,47	San Francisco Public Health Clinics	978 (MD and NP not provided separately)	Single visit	24 months	Monthly	Yes	1
58	WellMed, San Antonio	750				No	
57	Ohio Permanente Group	2650				No	
51	Palo Alto Medical Foundation		Single visit	12 months			
56	US Army Medical Clinic	1193				Yes	
43	Oregon Health & Science Univ	1227 MD, 848 NP	Single visit	36 months	Monthly	Yes	0.74
31,33,37	PC practices in Ontario	1300, 1800, 1400, 2000 in different practice models	Single visit	24 months		No	
44,46,48, 49	Univ Wisconsin Academic Practice	FP 2156, IM 1871, Peds 1750	Single visit	36 months	Monthly	No	0.5
34	3 Northern CA FQHCs		Two visits	18 months		Yes	
15,18	UCSF Health		Single visit	36 months	Monthly		
50,59	VA	1186	Single visit	24 months	Twice monthly	Mixed	0.75
13	Kaiser Permanente GA	2871				No	
16	18 PC Clinics Manitoba		Single visit	18 months			

© 2022 Mayo-Smith MF et al. *JAMA Network Open.*

Reference	Institution	Reported panel size per 1.0 CFTE MD	Criteria for panel assignment	Criteria for inactivation: duration without visit	Frequency of updating panel assignments	Advanced Practice Providers have own panel	Substitution ratio
18	UCLA		Single visit with prevention codes, or 2 visits in 3 years, or enrollment in HMO				
12,27,21	MetroHealth Cleveland	1146	Single visit	24 months	6 months		
19	Kaiser Permanente WA		Enrollment in health plan				
20,23	Mt Sinai NYC		Single visit	18 months			
35,38,39	Mayo Clinic, Rochester MN	2959	Contact with Mayo Clinic Rochester and live locally. PC visit past or future not required.	42 months (3.5 years)		Yes	
36	University Washington Medicine, Seattle		Single visit	36 months		Yes	
40	Alliance for Healthier Communities Clinics Ontario	1137 target	Single visit	36 months	Quarterly	Mixed	1
55	Cleveland Clinic OH					No	
22	MGMA members		Single visit	18 months			
45	Medical College of WI	MD 1000, APP 800	Single visit	18 months	Quarterly	Mixed	0.8
25	Duke		Two visits in 36 months and 1 in past 12 months	12 months	Annually		
26	Beth Israel Deaconess, Boston		Single visit	36 months	Annually		
28	Univ VA Charlottesville		Single visit	36 months	Quarterly		

© 2022 Mayo-Smith MF et al. *JAMA Network Open.*

Reference	Institution	Reported panel size per 1.0 CFTE MD	Criteria for panel assignment	Criteria for inactivation: duration without visit	Frequency of updating panel assignments	Advanced Practice Providers have own panel	Substitution ratio
**Empanelment Guides**							
29,30	Murray		Single visit	18 months	Monthly	Yes	
32	College FPs of Canada		Single visit	12-36 months. Best way is 18 months		No	
14	Safety Net Initiative		Two visits	18 months	Monthly		
17	AHRQ		Single visit	12 months	Monthly		
18	AMA		1-2 visits or health plan enrollment	18-36 months	Monthly or annually		
24	Weber		Single visit	12 months	Monthly		

© 2022 Mayo-Smith MF et al. *JAMA Network Open.*

eTable 3. Details of Adjustment for Clinical Full Time Employee (CFTE)

Reference	Institution	Panel Size	Study Type	Provider Capacity Adjusted for CFTE
8	George Washington University Health Plan and Clinics		case study	yes
9	Group Health Cooperative of Puget Sound	2126	panel size independent variable	yes
10	MGH	1029	develop predictive model to link patients to primary care provider	no
11	Group Health Seattle	1889	panel size independent variable	no
12	MetroHealth Cleveland	1146	panel size independent variable	no
16	Manitoba PC clinics		panel size independent variable	yes
19	Kaiser Permanente WA		panel size independent variable	yes
27	MetroHealth Cleveland		panel size independent variable	yes
31	PC Practices Ontario	1537	panel size independent variable	yes
34	PC practices in Northern California		ase Study	yes
37	PC practices in Ontario	445	panel size dependent variable	no
38	Mayo Clinic	934	panel size independent variable	no
39	Mayo Clinic	2959	panel size independent variable	yes
40	Alliance for Healthier Communities Clinics Ontario	1137	organizational handbook	yes
41	San Francisco Public Health Clinics	978	case study	yes
43	Oregon Health & Science Univ		panel size independent variable	no
44	Univ Wisconsin Academic Practice	FP 2156, IM 1871, Peds 1750	case study	yes
45	Medical College of Wisc	493	case study	no
58	WellMed, San Antonia	485	case study	no
59	VA	1186	panel size independent variable	yes

© 2022 Mayo-Smith MF et al. *JAMA Network Open.*

Reference	Institution	Panel Size	Study Type	Provider Capacity Adjusted for CFTE
60	Group Health Cooperative of Puget Sound	2136	panel size independent variable	yes
61	VA		panel size independent variable	yes
62	VA	811	panel size independent variable	no
63	VA	653	panel size independent variable	no
64	VA	610	panel size dependent variable	no
65	PCPS in Hudson Valley	597	panel size independent variable	no
66	Mayo Clinic	1025	panel size dependent variable	no
67	Mayo Clinic	1014	panel size independent variable	no

© 2022 Mayo-Smith MF et al. *JAMA Network Open.*

eTable 4. Statistical Analysis of Consequences of Adjustment for CFTE and Panel Size

Did adjust (yes)	Did not adjust (no)
2126	1029
1537	1889
2959	1146
1137	445
978	934
2156	493
1871	485
1750	811
1186	653
2136	610
1784	597
	1025
	1014
	856
T-Test	0.000757978
2 tailed T test t-value is 4.471 p=.00021 p<.001	

© 2022 Mayo-Smith MF et al. *JAMA Network Open.*

eTable 5. Correlation of Panel Size and Duration of Look-Back Period

Panel size	Look back (months)	Average
1000	18	1000
978	24	1299
1146	24	
1186	24	
1300	24	
1400	24	
1800	24	
2000	24	
1137	36	1628
1227	36	
1750	36	
1871	36	
2156	36	
2959	42	2959
Pearson correlation	0.602525118	
	p value =.02259 p<0.05	

© 2022 Mayo-Smith MF et al. *JAMA Network Open.*
